# Supplementary material for: Atacama Large Aperture Submillimeter Telescope (AtLAST) Science: Planetary and Cometary Atmospheres
Source: Open Res Eur. 2024 Dec 12;4:78. Originally published 2024 Apr 24. [Version 2] doi: 10.12688/openreseurope.17473.2 (PMC11297396; doi:10.12688/openreseurope.17473.2)
Supplement: Peer review history

# Reviewer report: Reviewer response for version 2

**DOI:** 10.21956/openreseurope.20100.r48657

**Published:** 2024-12-31

**Authors:** Andrey Baryshev

**Affiliations:** 1 University of Groningen, Groningen, The Netherlands

This article summarizes most important science cases in planetary research (solar system) which can be significantly advanced by a large aperture mm/submm facility. Arguments are given for the aperture size and other relevant specifications  in sensitivity, spectral resolution and focal plane array sizes. Atacama Large Aperture Submillimeter Telescope project has been used as template as far as telescope diameter and site are concerned.

 Very good overview is given of state of the art and existing questions in four major science cases have been presented: Giant planet atmospheres, Terrestrial-type atmospheres, Plumes from icy moons and  Comets. It has been convincingly argued, that access to large single dish telescope is required to advance the outlined scientific questions. 

 Reviewing conclusions that outlines provisional requirements to ATLAST instrumentation, I have a reservations on how a spectroscopic dynamic range of 10E5 on a bright background can be achieved within a reasonable observing time of a few hours. As planet is very bright and it will define the background level which will limit the spectroscopic sensitivity. A spectral linewidth with which this is achievable/required should be mentioned.

Is the case presented with sufficient detail to be useful for teaching or other practitioners?

Yes

Is the work clearly and accurately presented and does it cite the current literature?

Yes

If applicable, is the statistical analysis and its interpretation appropriate?

Not applicable

Are all the source data underlying the results available to ensure full reproducibility?

Yes

Are the conclusions drawn adequately supported by the results?

Yes

Is the background of the case’s history and progression described in sufficient detail?

Yes

Reviewer Expertise:

Astronomical instrumentation for submm/far infrared. In particular quantum limited heterodyne high spectral resolution systems.

I confirm that I have read this submission and believe that I have an appropriate level of expertise to confirm that it is of an acceptable scientific standard.

# Reviewer report: Reviewer response for version 2

**DOI:** 10.21956/openreseurope.20100.r48574

**Published:** 2024-12-17

**Authors:** Kaustubh Hakim

**Affiliations:** 1 Institute of Astronomy, KU Leuven, Leuven, Flanders, Belgium; 2 Royal Observatory of Belgium, KU Leuven, Leuven, Belgium

I recommend the article for indexing.

Is the case presented with sufficient detail to be useful for teaching or other practitioners?

Yes

Is the work clearly and accurately presented and does it cite the current literature?

Yes

If applicable, is the statistical analysis and its interpretation appropriate?

Yes

Are all the source data underlying the results available to ensure full reproducibility?

Yes

Are the conclusions drawn adequately supported by the results?

Yes

Is the background of the case’s history and progression described in sufficient detail?

Yes

Reviewer Expertise:

planetary science; astrobiology; geochemistry

I confirm that I have read this submission and believe that I have an appropriate level of expertise to confirm that it is of an acceptable scientific standard.

# Reviewer report: Reviewer response for version 1

**DOI:** 10.21956/openreseurope.18882.r42596

**Published:** 2024-08-14

**Authors:** Kaustubh Hakim

**Affiliations:** 1 Institute of Astronomy, KU Leuven, Leuven, Flanders, Belgium; 2 Royal Observatory of Belgium, KU Leuven, Leuven, Belgium

There is an urgent need for a larger aperture, multi-beam, single dish (sub)mm facility to improve sensitivity and dynamic range of weak molecular lines, to achieve high spatial resolution, and to perform instantaneous multi-beam spectral imaging. AtLAST is a planned unprecedented 50-m single-dish multi-beam facility with an instrument suite that accesses the 100-950 GHz frequency range. The manuscript highlights four major science cases including giant and terrestrial planet atmospheres, plumes of icy moons and comets. Overall, the case for AtLAST planetary science is comprehensive and well presented. I recommend approval with attention to the following very minor points.

 Science case i: paragraph 2: I would remove the phrase “and by extension, exoplanets with similar sizes and compositions” because there is no mention of specific cases for exoplanets. If this phrase were to be kept, I would recommend adding some exoplanet cases and providing information on whether the resolution would be sufficient to isolate exoplanet signals from those of their host stars.

 Science case ii: Titan: An opportunity is being missed here by not elaborating on the potential synergy between AtLAST and DragonFly.

 Science case ii: Mars: Can the Martian crustal magnetic field can be detected with AtLAST frequencies? If yes, it would be an exciting prospect. See, e.g., (Larsson et al.,2013)

 Science case iii: Can Triton be really studied with AtLAST given the poor numbers for Neptune in Table 1?

 Science case iii: Enceladus: The possibility of plume chemical evolution (e.g., HCN detection) of Enceladus is a tantalizing science case. Can mK sensitivity be maintained on short timescales at these frequencies?

Is the case presented with sufficient detail to be useful for teaching or other practitioners?

Yes

Is the work clearly and accurately presented and does it cite the current literature?

Yes

If applicable, is the statistical analysis and its interpretation appropriate?

Yes

Are all the source data underlying the results available to ensure full reproducibility?

Yes

Are the conclusions drawn adequately supported by the results?

Yes

Is the background of the case’s history and progression described in sufficient detail?

Yes

Reviewer Expertise:

planetary science; astrobiology; geochemistry

I confirm that I have read this submission and believe that I have an appropriate level of expertise to confirm that it is of an acceptable scientific standard.

## References

[1] : A method for remote sensing of weak planetary magnetic fields: Simulated application to Mars. *Geophysical Research Letters* .2013;40(19) : 10.1002/grl.50964 5014-5018 10.1002/grl.50964

# Response

**Published:** 2024-12-12

**Authors:** Martin Cordiner

**Affiliations:** Astrochemistry Laboratory, NASA Goddard Space Flight Center, Greenbelt, Maryland, USA

We thank the referee for their useful and insightful comments, which have helped significantly improve the manuscript. Our responses to their individual comments are given below, and are addressed in the latest, revised version of the article.   

 -----------------------------

**Reviewer Comment:**

 Science case i: paragraph 2: I would remove the phrase “and by extension, exoplanets with similar sizes and compositions” because there is no mention of specific cases for exoplanets. If this phrase were to be kept, I would recommend adding some exoplanet cases and providing information on whether the resolution would be sufficient to isolate exoplanet signals from those of their host stars.

**Author Response: **Unfortunately, it is unlikely that AtLAST would have sufficient spatial resolution (or sensitivity) to resolve the (sub)millimeter emission from even very large exoplanets --- note that ALMA, with its superior resolution, has recently detected accretion signatures in protoplanetary disks that imply the presence of forming planets, but ALMA has not observed any exoplanets directly. As mentioned in the introduction, the planets of our Solar System provide a key paradigm for the interpretation of a variety of exoplanet observations. Therefore, it is still useful to highlight the fact that a better understanding of our Solar System planets leads to improved understanding of exoplanets in general. 

 -----------------------------

**Reviewer Comment:**

 Science case ii: Titan: An opportunity is being missed here by not elaborating on the potential synergy between AtLAST and DragonFly.

**Author Response: **We now mention Dragonfly in the text for Science case ii. 

 -----------------------------

**Reviewer Comment:**

 Science case ii: Mars: Can the Martian crustal magnetic field can be detected with AtLAST frequencies? If yes, it would be an exciting prospect. See, e.g., (Larsson et al.,2013)

**Author Response: **Unfortunately, the 119 GHz O2 line investigated by Larsson et al. lies outside of the 3mm atmospheric transmission window, and therefore would not be observable by a ground-based telescope such as AtLAST

 -----------------------------

**Reviewer Comment:**

 Science case iii: Can Triton be really studied with AtLAST given the poor numbers for Neptune in Table 1?

**Author Response: **Similar to Titan and Enceladus, the fact that Triton has an angular diameter less than the size of the AtLAST beam does not preclude the possibility of detecting atmospheric or exospheric emission, as long as the telescope sensitivity is high enough.

 -----------------------------

**Reviewer Comment:**

 Science case iii: Enceladus: The possibility of plume chemical evolution (e.g., HCN detection) of Enceladus is a tantalizing science case. Can mK sensitivity be maintained on short timescales at these frequencies?

**Author Response:** We agree with the referee that temporally resolved studies of molecular abundance ratios would provide an interesting science case for Enceladus and other bodies. According to current estimates for the AtLAST sensitivity, the target spectral line sensitivity of ~1 mK per 1 km/s spectral bandwidth would be achievable in around 6 hours observing time (or a few mK in 1 h observing time). Although the intrinsic cadence of abundance variability in the Enceladus plume is unknown, at this nominal sensitivity, temporal abundance variations over a period of a few hours could be detectable. We suggest that the most conservative approach is to obtain secure molecular detections of the plume before planning a meaningful time-variability study.

# Reviewer report: Reviewer response for version 1

**DOI:** 10.21956/openreseurope.18882.r41371

**Published:** 2024-08-02

**Authors:** Takahiro Iino

**Affiliations:** 1 The University of Tokyo, Tokyo, Japan

Submillimeter radio wavelength observation technique has broaden our understandings of planetary environment and its origin. In particular, recent observational achievements by ALMA interferometer have played an important role thanks to its high spatial resolution and sensitivity. In turn, some technical limitations of interferometer have prevented us to obtain the precise observational parameter of solar system objects.

 This paper aims to summarize and introduce the solar system observational science possibly revealed by newly available wide-aperture single-dish submillimeter telescope, AtLast, which will dissolve the limitation of interferometric observation. In this manuscript, wide range of main solar system science theme have been covered by the professionals, and previous achievements in the fields are introduced properly. The needs for instrumental capability such as frequency coverage, dynamic range and time domain operation capability are described and summarized from scientific point of view. Despite the manuscript includes various scientific fields, citations of previous researches are well described and future perspective of AtLast is also appropriate and interesting. 

 This manuscript is important and helpful for providing possible scientific achievements to be addressed in 2030s with a new wide-aperture sub-millimeter telescope. In summary, I recommend that it for indexing after revising some minor points. Since this manuscript deal with a range of scientific theme, addition of some references may be helpful. In addition, consideration of revision of instrumental parameters may be needed. 

 --- Below are minor comments

 - In the Prior/existing instruments and results subsection in Introduction section, please consider to add (Rosenqvist et al., 1993)[ Ref-1] as the first detection paper of CO and HCN on Neptune achieved simultaneously with (Martenet et al.,1993),[Ref-2] as well as (Hesman et al.,2007) [Ref-3] as the implication of cometary CO supply on Neptune. 

 In addition, I suppose that (sub)millimeter observation campaign on Jupiter to investigate the aftermath of SL9 impact was a great advance on the solar system radio astronomy. Please consider to describe the campaign by citing (Moreno et al., 2003) [Ref-4], P&SS and relating materials. 

 - In the section of " *The Need for a Larger Aperture, Multi-Beam, Single* *Dish (Sub)Millimeter Facility*": ALMA's dynamic ranges are introduced here. Please consider to add technical reference of the described dynamic range value. 

 - In Dynamics subsection in Science case i section,  only J = 1-0 is listed as PH3 transition. On Jupiter, higher transitions may be better targets (please see Weisstein et al., 1996,[Ref-5] Icarus, for the radiative transfer analysis and FTS results). Please consider to add other transitions or to clarify the reason to choose J=1-0. 

 -  In Dynamics subsection in Science case i section, rounding of HCN rotational frequencies may not be correct.

 - In Chemistry subsection in Science case i section, frequency bands of nitriles are listed. Please consider to clarify the reason why the authors selected some specific frequency bands as the target bands. 

 - In Chemistry subsection in Science case i section, after " *With a spectroscopic dynamic range* *≳ 10* *4* *, measurement of more accurate isotopic ratios for...*". I suppose that the 13C carbon isotopic ratio has less scientific information than O, N and H, and measured already in some planetary atmosphere. please consider to add D/H in this list or to remove 13C. 

 - In science case iii section, the authors described that " *the peak antenna temperature for the*

*HCN J = 4 − 3 transition is calculated to be ∼ 25 mK*". However, considering 25K rotational temperature, rotational transition of HCN that exhibits maximum intensity at that environment may be J=3-2 or lower transition. Please consider to clarify the reasons why J=4-3 was chosen. 

 - In Science case iv section, as the authors described that " *Since comets are dynamic, time-variable objects...*", I believe that time-domain observation on comets is crucial. Please consider to add both short- and long-term changes observation results such as (Bockelee-Morvan et al.,2008) [Ref-6] of 17P/Holmes outburt and (Biver et al., 1997) [Ref-7] of C/1995 O1 monitoring result, respectively, for example.

Is the case presented with sufficient detail to be useful for teaching or other practitioners?

Yes

Is the work clearly and accurately presented and does it cite the current literature?

Yes

If applicable, is the statistical analysis and its interpretation appropriate?

Not applicable

Are all the source data underlying the results available to ensure full reproducibility?

Not applicable

Are the conclusions drawn adequately supported by the results?

Yes

Is the background of the case’s history and progression described in sufficient detail?

Yes

Reviewer Expertise:

(sub)millimeter observational study on the planetary atmsopheric chemistry and physics.

I confirm that I have read this submission and believe that I have an appropriate level of expertise to confirm that it is of an acceptable scientific standard.

## References

[1] : Millimeter-wave observations of Saturn, Uranus, and Neptune - CO and HCN on Neptune. *The Astrophysical Journal* .1992;392: 10.1086/186435 10.1086/186435

[2] : First observations of CO and HCN on Neptune and Uranus at millimeter wavelengths and the implications for atmospheric chemistry. *The Astrophysical Journal* .1993;406: 10.1086/172440 10.1086/172440

[3] : The abundance profile of CO in Neptune's atmosphere. *Icarus* .2007;186(2) : 10.1016/j.icarus.2006.08.025 342-353 10.1016/j.icarus.2006.08.025

[4] : Long-term evolution of CO, CS and HCN in Jupiter after the impacts of comet Shoemaker-Levy 9. *Planetary and Space Science* .2003;51(9-10) : 10.1016/S0032-0633(03)00072-2 591-611 10.1016/S0032-0633(03)00072-2

[5] : Submillimeter Line Search in Jupiter and Saturn. *Icarus* .1996;123(1) : 10.1006/icar.1996.0139 23-36 10.1006/icar.1996.0139

[6] : Large Excess of Heavy Nitrogen in Both Hydrogen Cyanide and Cyanogen from Comet 17P/Holmes. *The Astrophysical Journal* .2008;679(1) : 10.1086/588781 L49-L52 10.1086/588781

[7] : Evolution of the outgassing of comet Hale-Bopp (C/1995 O1) from radio observations. *Science* .1997;275(5308) : 10.1126/science.275.5308.1915 1915-8 10.1126/science.275.5308.1915 9072964

# Response

**Published:** 2024-12-12

**Authors:** Martin Cordiner

**Affiliations:** Astrochemistry Laboratory, NASA Goddard Space Flight Center, Greenbelt, Maryland, USA

We thank the referee for taking the time to review this article, and for their comments and suggestions for improvement, which we have incorporated into the revised version. Our responses to their individual comments are given below.  

**Reviewer Comments:**

 --- Below are minor comments

 - In the Prior/existing instruments and results subsection in Introduction section, please consider to add (Rosenqvist et al., 1993)[ Ref-1] as the first detection paper of CO and HCN on Neptune achieved simultaneously with (Martenet et al.,1993),[Ref-2] as well as (Hesman et al.,2007) [Ref-3] as the implication of cometary CO supply on Neptune.

**Author Response: ** Done.

 ---------------------------

**Reviewer Comments:**

 - In addition, I suppose that (sub)millimeter observation campaign on Jupiter to investigate the aftermath of SL9 impact was a great advance on the solar system radio astronomy. Please consider to describe the campaign by citing (Moreno et al., 2003) [Ref-4], P&SS and relating materials.

**Author Response: ** Done.

 ---------------------------

**Reviewer Comments:**

 - In the section of "The Need for a Larger Aperture, Multi-Beam, Single Dish (Sub)Millimeter Facility": ALMA's dynamic ranges are introduced here. Please consider to add technical reference of the described dynamic range value.

**Author Response: **Reference to the ALMA technical handbook added.

 ​​​​​​​---------------------------​​​​​​​

**Reviewer Comments:**

   - In Dynamics subsection in Science case i section,  only J = 1-0 is listed as PH3 transition. On Jupiter, higher transitions may be better targets (please see Weisstein et al., 1996,[Ref-5] Icarus, for the radiative transfer analysis and FTS results). Please consider to add other transitions or to clarify the reason to choose J=1-0. 

**Author Response: **We now also mention the PH3 800 GHz transition. Note that the 534 GHz transition is obscured by telluric H2O.

 ​​​​​​​---------------------------​​​​​​​

**Reviewer Comments:**

 -  In Dynamics subsection in Science case i section, rounding of HCN rotational frequencies may not be correct.

**Author Response: **Fixed. We also added the 177 GHz line.

 ​​​​​​​---------------------------​​​​​​​

**Reviewer Comments:**

 - In Chemistry subsection in Science case i section, frequency bands of nitriles are listed. Please consider to clarify the reason why the authors selected some specific frequency bands as the target bands. 

**Author Response: **There are too many transitions to mention for these, relatively more complex molecules, so a selection of representative transition frequencies was given. However, these were chosen in an ad-hoc manner. Considering the precise line strengths vary as a function of which planet we are looking at (and which region of the atmosphere), we now choose to no longer mention specific frequencies for these molecules.

 ​​​​​​​---------------------------​​​​​​​

**Reviewer Comments:**

 - In Chemistry subsection in Science case i section, after "With a spectroscopic dynamic range ≳ 104, measurement of more accurate isotopic ratios for...". I suppose that the 13C carbon isotopic ratio has less scientific information than O, N and H, and measured already in some planetary atmosphere. please consider to add D/H in this list or to remove 13C. 

**Author Response:** A number of unusual 13C isotopic ratio measurements have begun to emerge in the exoplanet and protoplanetary disk literature, so it seems worthwhile to continue to study it in the Solar System. We have now added D to the list of isotopes.

 ​​​​​​​--------------------------- 

**Reviewer Comments:**

 - In science case iii section, the authors described that "the peak antenna temperature for the

 HCN J = 4 − 3 transition is calculated to be ∼ 25 mK". However, considering 25K rotational temperature, rotational transition of HCN that exhibits maximum intensity at that environment may be J=3-2 or lower transition. Please consider to clarify the reasons why J=4-3 was chosen. 

**Author Response:** In choosing the J=4-3 transition, the balance of excitation and beam dilution factors were considered. The 3-2 and 4-3 lines actually have very similar opacities at 25 K, so the smaller beam size at 355 GHz leads to a higher beam-averaged antenna temperature.

 ​​​​​​​---------------------------​​​​​​​

**Reviewer Comments:**

 - In Science case iv section, as the authors described that "Since comets are dynamic, time-variable objects...", I believe that time-domain observation on comets is crucial. Please consider to add both short- and long-term changes observation results such as (Bockelee-Morvan et al.,2008) [Ref-6] of 17P/Holmes outburt and (Biver et al., 1997) [Ref-7] of C/1995 O1 monitoring result, respectively, for example.

**Author Response:** References have been added to support the statement. For comet Holmes, we cite Montalto et al. (2008) instead of Bockelee-Morvan et al.,2008, since it contains a more detailed description of the outburst event.

# Reviewer report: Reviewer response for version 1

**DOI:** 10.21956/openreseurope.18882.r41108

**Published:** 2024-06-24

**Authors:** Hideo Sagawa

**Affiliations:** 1 Kyoto Sangyo University, Kyoto, Japan

This paper describes the solar system science use cases for a large aperture single-dish submm telescope. Submm spectroscopic/mapping observations are quite powerful in solar system science. A large aperture single-dish can cover observation areas where existing ALMA and other telescopes are inadequate.

 The paper is clearly written, and I don't find any major concern for indexing.

 The authors provide a very clear overview of solar system science using submm-waves to date. This article is a very good introduction (with complete citations to important references in the field) for new researchers entering this field.  I thank the authors for this effort.

 ===

 The following are minor comments. I recognize that some of the comments are too technical to be described in this science use case paper, but I included them just for reference only.

 page 5

 - Introduction

 *1st paragraph: "100 - 950 GHz" -> Is there any special reason to define the upper boundary as 950 GHz? Perhaps, 1000 GHz can be accepted.

 *Last part of the 5th paragraph: The authors summarise existing interferometers, VLA, BIMA, SMA, and ALMA together in one sentence. I believe that ALMA is a revolutionary interferometer that has led to a significant improvement in observational performance compared to other interferometers. So, personally, I thought it would be good to distinguish references to ALMA, rather than describing it in the same sentence as BIMA and others, and to convey the fact that ALMA brought a new stages of interferometric observation.

 page 6

 * Right column (Improved sensitivity and dynamic range for molecular detections): I agree that the authors' arguments are very important points. Regarding the need for an extremely flat bandpass, is there any new technology planned to be implemented in AtLAST in the optics or receiver system? Or is the performance comparable to existing telescopes (e.g. JCMT)?

 As for standing waves, solar system objects are too bright, so I think we may need stricter standards than the usual astronomical sense.

 * Last sentence: "Cometary comae are highly diffuse and extended objects." -> Could you provide specific numbers here?

 page 7

 * Figure 1 and Table 1: I think there is considerable overlap in content. Table 1 could be removed, but I would leave it to the authors to decide.

 Also, it would be nice to have a figure or table to compare the spatial resolution with existing single-dish (JCMT) and interferometers (ALMA). However, if new figures and tables are added, the main text will also need some modifications.

 * Right column: "without severe beam dilution (particularly above ~800 GHz)." -> I would add a brief explanation about "beam dilution".

 * "Large planets such as Jupiter and Saturn are ... that are resolve out at long baselines." -> Could you provide specific numbers about how much the typical scale size to be resolved out is? Maybe adding some numbers from ALMA's maximum recoverable scale is a good idea.

 page 8

 * Left column (Instantaneous multi-beam spectral mapping): As an introduction to existing instruments, the authors could provide an example of the JCMT HARP.

 * Right column: "(1) small-scale vertical (radial) variations" -> Phrase "radial" seems odd for me...

 page 9

 * Left column: "...ALMA Band 3 observations (Figure 2, right panel)" -> I would add the information of wavelength for Band 3, as not all of readers are familiar with Band numbering.

 * Right column: "Wide bandwidth (>a few GHz)...so the deep tempereture and composition can be characterized, which complements observations in the IR down to ~1 bar." -> It would be very helpful if the authors can provide weighting functions of the listed submm lines (although I leave it to the authors to decide).

 * Right column: "Zonal wind speeds can also be inferred through infrared measurements of the thermal wind shear." -> Thermal wind equation is usually not applicable to equatorial region due to the low Coriolis force. Recently there is a new attempt to derive a modified thermal wind equation which is valid to the equator: Marcus et al. (2019) Icarus, 324, 198-223. https://doi.org/10.1016/j.icarus.2018.09.037

 The authors can cite this paper here. By the way, I did not think the current reference (Ref.79 future Giant planetary mission paper by Fletcher et al. 2020) necessarily appropriate.

 * Figure 2: The authors shows Jupiter maps taken by ALMA. In these maps, Jupiter's zone & belt features are clearly represented. On the other hand, somewhere in the main text, it was mentioned that ALMA is not very good at observing extended objects - which I agree with. In the case of this Jupiter maps, I thought it would be good to have a brief explanation of how such ALMA's shortcomings were dealt with.

 page 10

 * Right column (at the 1st sentence of "Chemistry"): ""non-equilibrium"" -> Please add more explanation, as it is not sure what you mean by non-equilibrium with respect to what.

 page 11

 * Left column, 1st sentence: "To perform these investigations requires wideband (> 16 GHz), ..." -> I thought that if there were some kind of simulated spectrum plot that shows very broad absorption, it would make the specificity of the need for a bandwidth > 16 GHz more compelling.

 Alternatively, it would be good to describe some specific numbers on how broad the Neptune CO line (cited in this paragraph) actually are in terms of the broad absorptioon wings.

 * Right column (1st paragraph of Science case II): there is an unnecessary hyphen in "spectro-scopic".

 * Right column: Venus is usually close to the Sun, and its closeness is always problem for ground-based observations. Does AtLAST have any sun avoidance elongation limitation? Or, can it observe an object very close to the Sun?

 * Right column: "Mapping ... Venus with strong transitions (such as SO2, SO, and CO)" --> I am a little reluctant to say SO2 or SO as a strong line. At the very least, I think it is by far the weakest compared to CO, no?

 page 12

 * Left column (2nd paragraph): "The Venus Express and Akatsuki ... detected ... SO2 and CO abundances..." -> Maybe the authors can put some references here (is there any work about long term variation of CO?).

 * Left column (last paragraph): "... ground-based measurements ... will be required to contextualize in situ data from ... DAVINCI+, VERITAS, and EnVision." -> Remove "in situ" because DAVINCI is the only in-situ probe mission and the other two, VERITAS and EnVision, do not carry any in-situ atmospheric sensors.

 * Right column (1st paragraph): "...the comparative weakness of the mm-wave absorption lines..." -> I think "mm" should be "submm" or "(sub)mm".

 * Right column (last paragraph): "Discrepancies between the observed wind ... predicted by state-of-the-art climate models ..." -> This sentence is discussing about the Martian atmosphere at very high altitude of 140 - 240 km. It appears a little strange to call a model of such high altitude atmosphere a climate model. I think it is better to call it "a global ionosphere-thermosphere model" or simply "a general circulation model".

 page 13

 * Left column first paragraph: "As shown by 113, comparison of ground-based Mars CO mapping... (see Figure 5) indicates significant discrepancies in the wind speeds and their spatial structure..." -> Current Fig.5 shows the spectral mapping of CO (1-0), and from this figure alone, it is difficult to determine the discrepancy in the wind speed field as described in the text. I think it would be better to revise the sentence in the text slightly or revise the figure to one that much clearly shows the difference from the model regarding winds.

 * Although the authors do not specifically mention it, it can also be pointed out that one of the most interesting aspects of (sub)mm-wave observations of Mars is that the optical thickness of Martian dust is nearly negligible. Indeed, there are some unique past studies, such as Gurwell et al. (2005) Icarus, 175, 23-31

  https://doi.org/10.1016/j.icarus.2004.10.009 .

 page 14

 * Left column (middle area): "...N2/C2H4 and HCN/C2H3 ref127;)." -> Unnecessary ; is inserted.

 * Left colum last paragraph: "To avoid Saturn and exclude it from the 2nd and 3rd sidelobes of the primary beam..." -> I agree that this is a very important point. In fact, in past Herschel observations of Ganymede, the team detected a strong H2O absorption line (much to the excitement of the team members), but upon closer examination, they found that the sidelobes of Herschel's primary beam were partially overlapping with Jupiter. If interested, check the conference presentation named "Detection and Characterisation of Ganymede's and Callisto's Water Atmospheres" by Hartogh et al. (2013) in "The Universe Explored by Herschel".

  https://www.cosmos.esa.int/web/herschel/the-universe-explored-by-herschel

 page 15

 * Right column: "...in Figure 6 will require milli-Kelvin sensitivity..." -> I would use "~1 mK sensitivity" to unify notation with other parts.

 page 17

 * Summary

 For the solar system, especially planetary atmospheres, capability of long-term, continuous (monitoring) observations is important. ALMA has strict limitations on the duplicated observation proposals, and it is difficult to propose long-term continuous observations over multiple cycles. Does AtLAST anticipate such long-term observation proposals?

Is the case presented with sufficient detail to be useful for teaching or other practitioners?

Yes

Is the work clearly and accurately presented and does it cite the current literature?

Yes

If applicable, is the statistical analysis and its interpretation appropriate?

Not applicable

Are all the source data underlying the results available to ensure full reproducibility?

Not applicable

Are the conclusions drawn adequately supported by the results?

Yes

Is the background of the case’s history and progression described in sufficient detail?

Yes

Reviewer Expertise:

planetary science; (sub)mm and infrared observations; radiative transfer in planetary atmospheres

I confirm that I have read this submission and believe that I have an appropriate level of expertise to confirm that it is of an acceptable scientific standard.

# Response

**Published:** 2024-12-12

**Authors:** Martin Cordiner

**Affiliations:** Astrochemistry Laboratory, NASA Goddard Space Flight Center, Greenbelt, Maryland, USA

We thank the referee for their useful and insightful comments, which have helped significantly improve the manuscript. Our responses to their individual comments are given below, and are addressed in the latest, revised version of the article.

 page 5

 - Introduction

 *1st paragraph: "100 - 950 GHz" -> Is there any special reason to define the upper boundary as 950 GHz? Perhaps, 1000 GHz can be accepted.

**Author Response: **Indeed, the upper end of the submillimeter band lies at THz frequencies so we have modified the text accordingly. 

 *Last part of the 5th paragraph: The authors summarise existing interferometers, VLA, BIMA, SMA, and ALMA together in one sentence. I believe that ALMA is a revolutionary interferometer that has led to a significant improvement in observational performance compared to other interferometers. So, personally, I thought it would be good to distinguish references to ALMA, rather than describing it in the same sentence as BIMA and others, and to convey the fact that ALMA brought a new stages of interferometric observation.

**Author Response: **Agree. Text modified accordingly.

 page 6

 * Right column (Improved sensitivity and dynamic range for molecular detections): I agree that the authors' arguments are very important points. Regarding the need for an extremely flat bandpass, is there any new technology planned to be implemented in AtLAST in the optics or receiver system? Or is the performance comparable to existing telescopes (e.g. JCMT)?

 As for standing waves, solar system objects are too bright, so I think we may need stricter standards than the usual astronomical sense.

**Author Response: **We thank the referee for reiterating the importance of an extremely flat spectral bandpass.  We therefore recommend in our article that mitigation of reflections and standing waves in the optics be factored into the AtLAST telescope and receiver design. 

 * Last sentence: "Cometary comae are highly diffuse and extended objects." -> Could you provide specific numbers here?

**Author Response: ** Done. 

 page 7

 * Figure 1 and Table 1: I think there is considerable overlap in content. Table 1 could be removed, but I would leave it to the authors to decide.

 Also, it would be nice to have a figure or table to compare the spatial resolution with existing single-dish (JCMT) and interferometers (ALMA). However, if new figures and tables are added, the main text will also need some modifications.

**Author Response: ** Table 1 clarifies the frequency dependence of the angular resolution, and helps assist in the interpretation of Figure 1, so we prefer to keep it. 

 * Right column: "without severe beam dilution (particularly above ~800 GHz)." -> I would add a brief explanation about "beam dilution".

**Author Response: **Done.   

 * "Large planets such as Jupiter and Saturn are ... that are resolve out at long baselines." -> Could you provide specific numbers about how much the typical scale size to be resolved out is? Maybe adding some numbers from ALMA's maximum recoverable scale is a good idea. **Author Response: ** Done.

 page 8

 * Left column (Instantaneous multi-beam spectral mapping): As an introduction to existing instruments, the authors could provide an example of the JCMT HARP.

**Author Response: **A reference to the JCMT HARP study of comet R2/PanSTARRS by Cordiner et al. (2022) has been added.

 * Right column: "(1) small-scale vertical (radial) variations" -> Phrase "radial" seems odd for me...

**Author Response: ** It is odd nomenclature for a planetary scientist, yet "vertical" is also an odd term for an astrophysicist, so the clarification seems useful, given the broad utility of AtLAST in the astrophysics community.

 page 9

 * Left column: "...ALMA Band 3 observations (Figure 2, right panel)" -> I would add the information of wavelength for Band 3, as not all of readers are familiar with Band numbering. **Author Response: **Done.

 * Right column: "Wide bandwidth (>a few GHz)...so the deep tempereture and composition can be characterized, which complements observations in the IR down to ~1 bar." -> It would be very helpful if the authors can provide weighting functions of the listed submm lines (although I leave it to the authors to decide).

**Author Response: TBD**

 * Right column: "Zonal wind speeds can also be inferred through infrared measurements of the thermal wind shear." -> Thermal wind equation is usually not applicable to equatorial region due to the low Coriolis force. Recently there is a new attempt to derive a modified thermal wind equation which is valid to the equator: Marcus et al. (2019) Icarus, 324, 198-223. https://doi.org/10.1016/j.icarus.2018.09.037

 The authors can cite this paper here. By the way, I did not think the current reference (Ref.79 future Giant planetary mission paper by Fletcher et al. 2020) necessarily appropriate.

**Author Response:** The correct reference to a different paper by Fletcher et al. (2020) has now been added.

 * Figure 2: The authors shows Jupiter maps taken by ALMA. In these maps, Jupiter's zone & belt features are clearly represented. On the other hand, somewhere in the main text, it was mentioned that ALMA is not very good at observing extended objects - which I agree with. In the case of this Jupiter maps, I thought it would be good to have a brief explanation of how such ALMA's shortcomings were dealt with.

**Author Response:** We thank the referee for pointing out this apparent contradiction and have added a note of explanation to the Figure caption regarding the lack of short spacing data, which is, in fact, not needed for such "longitudinally smeared" images.

 page 10

 * Right column (at the 1st sentence of "Chemistry"): ""non-equilibrium"" -> Please add more explanation, as it is not sure what you mean by non-equilibrium with respect to what.

**Author Response:** An explanation of the concept of non-equilibrium chemistry is beyond the scope of our article. We refer the reader to the references given at the end of the following sentence if they wish to learn more.

 page 11

 * Left column, 1st sentence: "To perform these investigations requires wideband (> 16 GHz), ..." -> I thought that if there were some kind of simulated spectrum plot that shows very broad absorption, it would make the specificity of the need for a bandwidth > 16 GHz more compelling.

 Alternatively, it would be good to describe some specific numbers on how broad the Neptune CO line (cited in this paragraph) actually are in terms of the broad absorptioon wings.

**Author Response:** We have clarified the Neptune CO line morphology and added additional references to Rosenqvist et al. (1992), Lellouch et al. (2005) and Hesman et al. (2007).

 * Right column (1st paragraph of Science case II): there is an unnecessary hyphen in "spectro-scopic".

**Author Response: **We refer this issue to the journal typesetters.

 * Right column: Venus is usually close to the Sun, and its closeness is always problem for ground-based observations. Does AtLAST have any sun avoidance elongation limitation? Or, can it observe an object very close to the Sun?

**Author Response: **As mentioned in the article, AtLAST is expected to allow observations of near-sun targets including Mercury and Venus, which will necessitate pointing of the telescope at solar elongations ~10-30 degrees. We have added a new subsection to the Summary near the end of the article to clarify these requirements.  Solar observations are also in the current AtLAST science portfolio (see https://arxiv.org/pdf/2403.00920).

 * Right column: "Mapping ... Venus with strong transitions (such as SO2, SO, and CO)" --> I am a little reluctant to say SO2 or SO as a strong line. At the very least, I think it is by far the weakest compared to CO, no? 

**Author Response:** We thank the referee for pointing this out, and have revised the text accordingly.

 page 12

 * Left column (2nd paragraph): "The Venus Express and Akatsuki ... detected ... SO2 and CO abundances..." -> Maybe the authors can put some references here (is there any work about long term variation of CO?).

**Author Response:** The required information can be found in the review by Marcq et al. (2018). Reference added.

 * Left column (last paragraph): "... ground-based measurements ... will be required to contextualize in situ data from ... DAVINCI+, VERITAS, and EnVision." -> Remove "in situ" because DAVINCI is the only in-situ probe mission and the other two, VERITAS and EnVision, do not carry any in-situ atmospheric sensors.

**Author Response:** We have added "and orbiting" to clarify the situation.

 * Right column (1st paragraph): "...the comparative weakness of the mm-wave absorption lines..." -> I think "mm" should be "submm" or "(sub)mm".

**Author Response: **Done.

 * Right column (last paragraph): "Discrepancies between the observed wind ... predicted by state-of-the-art climate models ..." -> This sentence is discussing about the Martian atmosphere at very high altitude of 140 - 240 km. It appears a little strange to call a model of such high altitude atmosphere a climate model. I think it is better to call it "a global ionosphere-thermosphere model" or simply "a general circulation model".

**Author Response:** Done.

   page 13

 * Left column first paragraph: "As shown by 113, comparison of ground-based Mars CO mapping... (see Figure 5) indicates significant discrepancies in the wind speeds and their spatial structure..." -> Current Fig.5 shows the spectral mapping of CO (1-0), and from this figure alone, it is difficult to determine the discrepancy in the wind speed field as described in the text. I think it would be better to revise the sentence in the text slightly or revise the figure to one that much clearly shows the difference from the model regarding winds.

**Author Response:** Done.

 * Although the authors do not specifically mention it, it can also be pointed out that one of the most interesting aspects of (sub)mm-wave observations of Mars is that the optical thickness of Martian dust is nearly negligible. Indeed, there are some unique past studies, such as Gurwell et al. (2005) Icarus, 175, 23-31

  https://doi.org/10.1016/j.icarus.2004.10.009 .

**Author Response: **Reference to Gurwell et al. (2005) added.

 page 14

 * Left column (middle area): "...N2/C2H4 and HCN/C2H3 ref127;)." -> Unnecessary ; is inserted.

**Author Response: **Fixed.

 * Left colum last paragraph: "To avoid Saturn and exclude it from the 2nd and 3rd sidelobes of the primary beam..." -> I agree that this is a very important point. In fact, in past Herschel observations of Ganymede, the team detected a strong H2O absorption line (much to the excitement of the team members), but upon closer examination, they found that the sidelobes of Herschel's primary beam were partially overlapping with Jupiter. If interested, check the conference presentation named "Detection and Characterisation of Ganymede's and Callisto's Water Atmospheres" by Hartogh et al. (2013) in "The Universe Explored by Herschel".

  https://www.cosmos.esa.int/web/herschel/the-universe-explored-by-herschel

 page 15

 * Right column: "...in Figure 6 will require milli-Kelvin sensitivity..." -> I would use "~1 mK sensitivity" to unify notation with other parts.

**Author Response: ** Done.

 page 17

 * Summary

 For the solar system, especially planetary atmospheres, capability of long-term, continuous (monitoring) observations is important. ALMA has strict limitations on the duplicated observation proposals, and it is difficult to propose long-term continuous observations over multiple cycles. Does AtLAST anticipate such long-term observation proposals?

**Author Response:** A sentence on the requirement for long-term observations spanning several years has been added at the end of the Summary.
